# Loss of digestive organ expansion factor (*Diexf)* reveals an essential role during murine embryonic development that is independent of p53

**DOI:** 10.18632/oncotarget.22087

**Published:** 2017-10-26

**Authors:** Neeraj K. Aryal, Amanda R. Wasylishen, Vinod Pant, Maurisa Riley-Croce, Guillermina Lozano

**Affiliations:** ^1^ Department of Genetics, The University of Texas MD Anderson Cancer Center, Houston, TX 77030, USA; ^2^ Genes and Development Program, The University of Texas MD Anderson Cancer Center, UT Health Graduate School of Biomedical Sciences, Houston, TX 77030, USA

**Keywords:** CRISPR/Cas9, mouse model, ribosome small subunit processome, Mdm4 co-amplification, Def-Capn3 nucleolar pathway

## Abstract

Increased levels of inhibitors of the p53 tumor suppressor such as Mdm2 and Mdm4 drive tumor development and thus serve as targets for therapeutic intervention. Recently, digestive organ expansion factor (Diexf) has been identified as a novel inhibitor of p53 in zebrafish. Here, we address the potential role of Diexf as a regulator of the p53 pathway in mammals by generating *Diexf* knockout mice. We demonstrate that, similar to *Mdm2* and *Mdm4*, homozygous deletion of *Diexf* is embryonic lethal. However, unlike in *Mdm2* and *Mdm4* mice, loss of p53 does not rescue this phenotype. Moreover, *Diexf* heterozygous animals are not sensitive to sub-lethal ionizing radiation. Thus, we conclude that *Diexf* is an essential developmental gene in the mouse, but is not a significant regulator of the p53 pathway during development or in response to ionizing radiation.

## INTRODUCTION

The guardian of the genome, p53, is a tumor suppressor and transcription factor that initiates cell cycle arrest and apoptosis in response to stress or insult and thereby prevents damaged cells from proliferating. At the same time, p53 transcriptional activity is tightly regulated in normal cells to prevent inappropriate activation of downstream anti-proliferative or cell death programs [1]. The two most prominent p53 inhibitors are Mdm2 and Mdm4 [2]. Mouse models have demonstrated that both Mdm2 and Mdm4 have essential roles in embryonic development through inhibition of p53 activity. Homozygous loss of either gene is embryonic lethal, a phenotype that is completely rescued by the concomitant deletion of *p53* [3–5]. Additionally, transgenic overexpression of either is sufficient to promote tumorigenesis [6, 7]. In human cancers, increased levels of MDM2 and MDM4 abrogate the need for *TP53* alterations [8]. However, many cancers retain wild type *TP53* without alterations in known TP53 inhibitors suggesting that novel mechanisms of pathway inhibition remain to be identified [8].

Recently, digestive organ expansion factor (*Diexf*), was identified as a novel inhibitor of p53 in zebrafish [9–14]. In addition, in zebrafish and human cell lines, Def/DIEXF recruits Calpain3 in the nucleolus to regulate p53 stability through the Def-Capn3 pathway [15]. *DIEXF* is an evolutionarily conserved gene that was first identified in zebrafish and shown to be essential for expansion of digestive organs [9]. It is located on chromosome 1 in both human and mouse genomes coding for a 756 (772 in mouse) amino acid protein. DIEXF does not have any known functional domains except a glutamic acid repeat region in the amino-terminus which has been linked to RNA-processing [16]. Recent works in yeast, Arabidopsis, and zebrafish have demonstrated that Def is essential for pre-rRNA processing [16–18].

Loss of *Def* in zebrafish selectively up-regulates the expression of ∆113p53 isoform, transcribed from an alternative promoter in intron 4, in digestive organs [9, 19, 20]. *Def*-null fish overexpress ∆113p53 which results in increased p53 target gene expression and induction of cell cycle arrest in the digestive organs. This leads to under-expanded digestive organs and lethality by 8–11 days after fertilization [9]. Knock-down of both full-length p53 and ∆113p53 levels partially rescues the mutant phenotype indicating that Def acts as a negative regulator of p53 in zebrafish.

This study addresses the role of Diexf as a potential regulator of the p53 pathway in mammals. We demonstrate that *DIEXF* is amplified in many human cancers, and amplification is mutually exclusive with TP53 alterations in breast cancer. To directly evaluate the function of Diexf *in vivo*, we generated two independent knockout alleles in the mouse using the CRISPR/Cas9 technology. While heterozygous mice were normal, homozygous deletion of *Diexf* results in peri-implantation lethality. Concomitant deletion of *p53* did not rescue the embryonic lethality in *Diexf* knockout animals. We additionally demonstrate that *Diexf* heterozygosity does not affect the radiation sensitivity of adult mice as observed in *Mdm2* and *Mdm4* heterozygous mice. Combined, these data suggest that while *Diexf* is an essential developmental gene in the mouse, it is not a significant regulator of the p53 pathway.

## RESULTS

### *DIEXF* is amplified in human cancers with an inverse correlation to *TP53* alterations

**Figure 1:**
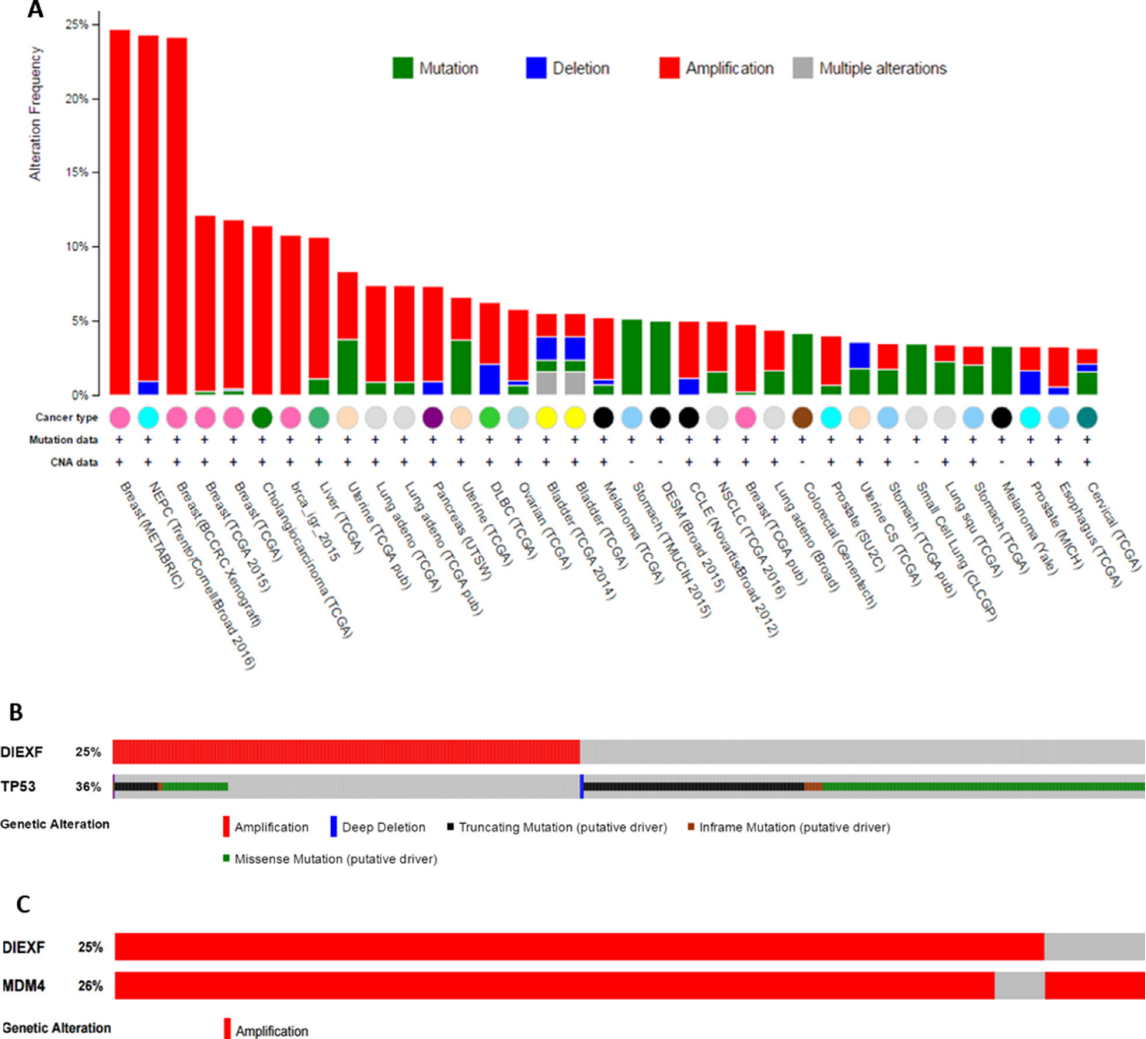
*DIEXF* amplification in human cancers (**A**) Human cancer datasets from cBioPortal showing *DIEXF* alterations in human cancers. (**B**) A breast cancer dataset (METABRIC) of 2051 patients shows *DIEXF* amplification and *TP53* alterations. There is a significant inverse correlation between *DIEXF* amplification and *TP53* alterations (*P* < 0.001). Only cases with alteration in queried gene are shown. (**C**) A breast cancer dataset (METABRIC) of 2051 patients shows *DIEXF* and *MDM4* co-amplifications. Only cases with amplification of at least one gene are shown.

*Diexf* is an evolutionarily conserved gene from yeast to humans, and recent studies in zebrafish have suggested that the Diexf protein is a negative regulator of p53 [9, 15]. In many different human cancers with wild type *TP53*, other *TP53* inhibitors such as *MDM2* and *MDM4* are frequently amplified or overexpressed [8, 21]. To ascribe a role of DIEXF in human cancers, we exploited the data from cBioPortal and examined whether a similar inverse correlation between *TP53* and *DIEXF* exists [22, 23]. We observed that *DIEXF* is indeed amplified in many human cancers (Figure 1A) including > 10% of the breast cancers, neuroendocrine prostate cancers (NEPCs), and cholangiosarcomas. In a breast cancer dataset of 2051 cases [24], 36% of total cases (*n* = 734) had *TP53* alterations and 25% (*n* = 513) had Diexf amplification, but only 6% (*n* = 134) had both (Figure 1B). A significant mutual exclusivity (*P* < 0.001) was present between *DIEXF* amplification and *TP53* alterations. Significant mutual exclusivity was also observed in other breast cancer datasets in Figure 1A. These results support the hypothesis that DIEXF is a negative regulator of the p53 pathway. However, it is important to note that *DIEXF* is located on chromosome 1q in close proximity (5.3Mbp) to the *MDM4* locus. It is thus possible that *DIEXF* amplification is a consequence of co-amplification with *MDM4*. When we analyzed the same breast cancer datasets, we observed that amplification of *DIEXF* and *MDM4* significantly overlapped (*P* < 0.001, Figure 1C). Similarly, when we examined other tumor types with *DIEXF* amplification, we found significant overlap with *MDM4* amplification. We did not find any cancer where *DIEXF* amplification was significantly higher than *MDM4* amplification. However, cases where either *MDM4* or *DIEXF* amplification occurred independent of each other were also present in all tumor types examined.

### Generation of *Diexf* knock-out mice

**Figure 2:**
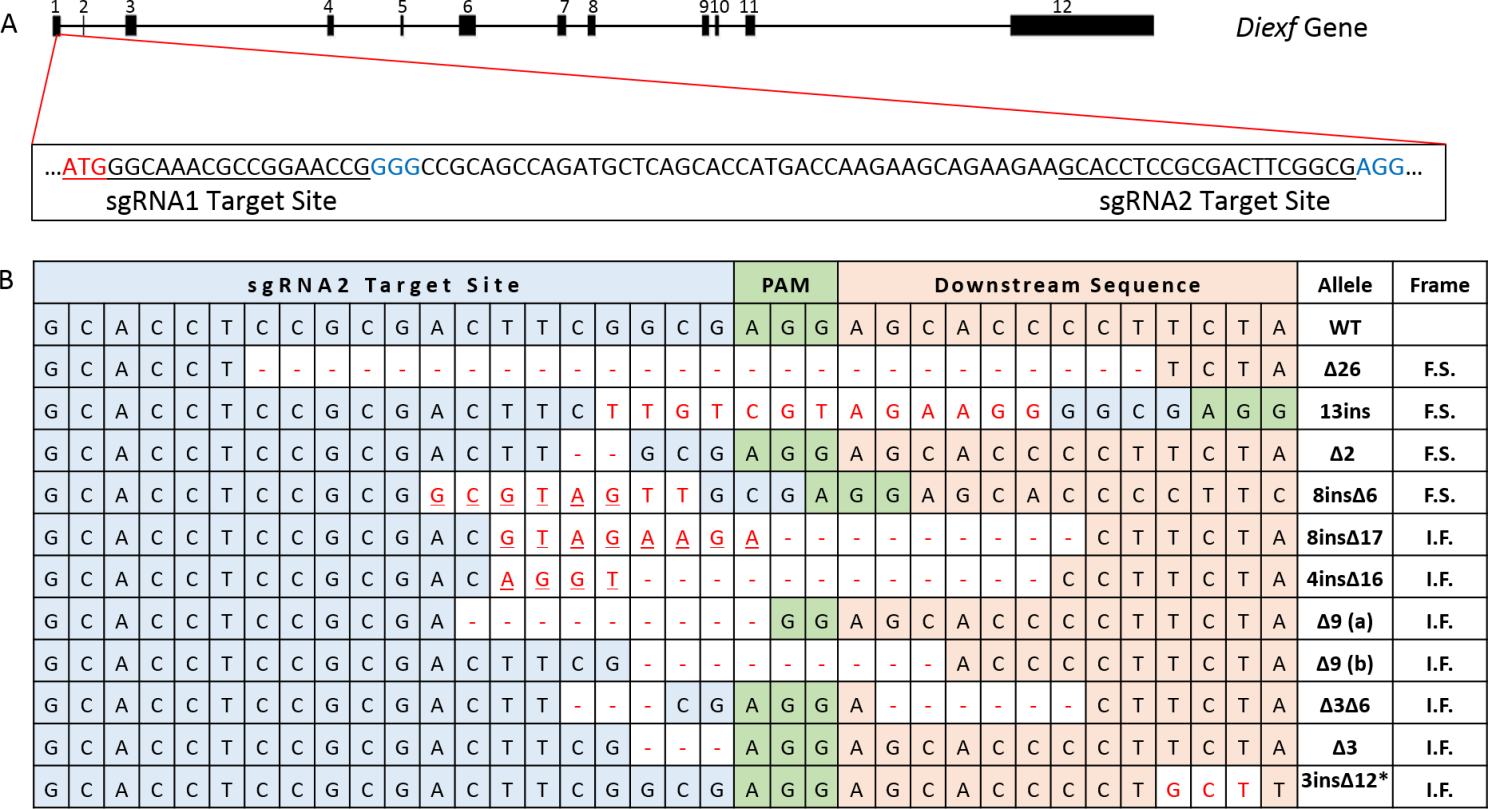
*Diexf* targeting using CRISPR/Cas9 system (**A**) Genomic map of *Diexf* gene with 12 exons highlighting the sequence of exon 1 with the two target sites. The size of exons and introns are proportional to their width in the map. Translational start site (start codon) is in red, target sequence is underlined, and Protospacer adjacent motif (PAM) sequence is in blue. (**B**) Details of all alleles generated with alterations. Target site 2 is in blue cells, and alterations (deletion [-] or insertion) are in red. Underlined bold letters represent deletion and insertion at the same position. I.F., in-frame mutation; F.S., frame-shift mutation; ^*^, an additional 12 base deletion (not shown) in this allele is 156 bases downstream of the 3 base insertion site.

To directly evaluate the function of Diexf and its potential role in regulation of the p53 pathway *in vivo*, we generated a *Diexf* deletion in mice using CRISPR/Cas9 technology. *Diexf* is a 26 Kbp gene with 12 exons that code for a 772 amino acid protein (89 KDa) in mice. Exon 1 is 229 base pairs and contains the translational start site. We selected two sequences in Exon 1 downstream of the start codon to target with specific sgRNAs and the *spCas9* endonuclease (Figure 2A) respectively. The two target sequences are located at the translational start site (bases 123–142) and further downstream (bases 188–207). Both sgRNAs have very few candidate off-target sites with scores of 98 and 93 out of 100 respectively per crispr.mit.edu analysis tool (Supplementary Table 1). When both sgRNAs and *spCas9* endonuclease were used, only mice with disruption of the *Diexf* alleles at site 2 were obtained. Multiple alleles with both in-frame and frame-shift mutations were generated (Figure 2B). Of 16 animals born, 14 had alterations in one or both alleles of the *Diexf* gene (Table 1). Even though both alleles were targeted in 6 mice, no mice were born with two frame-shift alleles suggesting that homozygous loss may be lethal (Table 1). We selected two alleles with frame shift mutations for further experiments. The first allele *Diexf*^∆26^ had 26 base pairs deleted (bases 194–219 in exon 1) resulting in a premature stop signal after 85 amino acids (23 endogenous and 62 novel amino acids). The second allele *Diexf*^13ins^ had a 13 base pair insertion between bases 200–201 in exon 1 resulting in premature stop after 28 amino acids (26 endogenous and 2 novel amino acids).

**Table 1:** Genotype of *Diexf* alleles in all mice generated by CRISPR/Cas9 targeting

Mouse #	Genotype	
	Allele 1	Allele 2
1	13ins	∆9 (a)
2	∆26	∆9 (a)
3	∆26	WT
4^*^	3ins∆12	∆9 (b) and ∆26
5^*^	∆2	4ins∆16 and ∆9 (a)
6	∆3∆6	8ins∆6
7	∆9 (a)	∆9 (a)
8^*^	∆9 (a)	∆3 and WT
9^*^	∆9 (a)	8ins∆17 and WT
10–14 (***n =*** 5)	∆9 (a)	WT
15–16 (***n =*** 2)	WT	WT

^*^, Mosaics. Of 16 mice, 14 had targeting in at least one allele. 6 animals had bi-allelic targeting (animals with no wild type allele, 2/6 mosaics). At least one allele in all animals are wild type or in-frame. ∆; deletion, ins; insertion, WT; wild type, (a) and (b); different ∆9 alleles.

To rule out the possibility of unintended mutations being incorporated into the genome by the CRISPR/Cas9 system, we evaluated off-target cleavage at the top six sites for sgRNA2 and top four sites for sgRNA1 based on scoring by crispr.mit.edu analysis tool (Supplementary Table 1). We did not observe any off-target mutations in these mice. *Diexf*^∆26^ and *Diexf*^13ins^ mice were crossed with wild type animals for three generations to further mitigate the potential of any off-target mutation being carried over to the experimental cohort.

### *Diexf* null embryos are peri-implantation lethal

The two selected lines of *Diexf* knock-out alleles with frame-shift truncating mutations were used to evaluate if homozygous null animals were viable. For each line, we inter-crossed heterozygous animals and genotyped progeny at the time of weaning. Of 22 pups born from 3 litters in the *Diexf*^13ins^ line, 7 animals were wild type, 15 were heterozygous mutants, and no animals were homozygous mutants (Table 2). Similarly, of 48 pups born from 7 litters in the *Diexf*^∆26^ line; 19 animals were wild type, 29 were heterozygous, and none were homozygous mutants (Table 2). The deviations from the expected Mendelian ratios were significant in both lines (*p =* 0.02 and *p =* 0.0001 respectively). Failure to generate any viable homozygous knock-out animals clearly demonstrates an essential role of Diexf during embryonic development in mice.

**Table 2:** Homozygous deletion of *Diexf* is embryonic lethal at peri-implantation stage (E4.5-E5.5) in mice

*Diexf*^+/−^ X *Diexf*^+/−^ : Observed (Expected)					
Allele	Age	*Diexf*^+/+^	*Diexf*^+/−^	*Diexf*^−/−^	Empty Decidua
*Diexf*^*13ins*^	P21	7 (6)	15 (11)	0 (6)	-
*Diexf*^*∆26*^	P21	19 (12)	29 (24)	0 (12)	-
*Diexf*^*∆26*^	E14.5	4 (3)	9 (7)	0 (3)	0
*Diexf*^*∆26*^	E9.5	4 (4)	10 (7)	0 (4)	4
*Diexf*^*∆26*^	E7.5	9 (6)	15 (12)	0 (6)	6

Heterozygous crosses between *Diexf*^+/−^ mice (two lines) and observed vs expected numbers for all genotype at various stages. P21 = 21 days after birth. E = embryonic day. All expected numbers are rounded to the nearest integer.

In order to determine the timing of the lethality of homozygous mutants, we harvested and genotyped the embryos from *Diexf*^+/∆26^ heterozygous crosses at various developmental time points. We screened 13 embryos from 2 females at E14.5 and did not find any homozygous mutants (Table 2). At E9.5, we screened 18 embryos from 3 females and observed 4 wild type, 10 heterozygous mutant embryos, and 4 empty decidua. Similarly, of 30 embryos from 4 females at E7.5, we observed 6 empty decidua while the remaining embryos were either wild type (*n* = 9) or heterozygous mutants (*n* = 15). The finding of empty decidua demonstrated that mutant embryos implanted but died soon thereafter and were likely resorbed by E7.5 [25]. This clearly demonstrates that the *Diexf* null embryos are peri-implantation lethal at E4.5-E5.5 stages. To further evaluate the viability of mutant embryos at pre-implantation stage, we flushed out blastocysts from heterozygous crosses, observed the morphology under microscope, and genotyped them. We did not observe obvious defects in dozens of blastocysts examined, and genotyping of 8 random blastocysts confirmed presence of homozygous mutants (Supplementary Figure 1). Combined, these results establish *Diexf* as an essential gene for early embryonic development.

### Embryonic lethality of *Diexf*^*-/-*^ mice is p53-independent

Early embryonic lethality occurs in knock-out mouse models of the p53 inhibitors Mdm2 and Mdm4. Lethality in these models is p53-dependent as the phenotype is rescued upon concomitant deletion of *p53*. Studies in zebrafish suggest that Diexf is a potential negative regulator of p53, and embryonic lethality of *Def*-null fish is partially p53-dependent. In order to address if embryonic lethality of *Diexf*-null mice is also p53-dependent, we crossed heterozygous animals carrying *the Diexf*^∆26^ null allele with *p53*^-/-^ mice. The resulting *Diexf*^+/∆26^*; p53*^+/-^ double heterozygous mice were inter-crossed and progeny were genotyped at weaning. We screened 108 animals from 15 litters and observed no *Diexf*^∆26/∆26^ homozygous mutants irrespective of *p53* status (Table 3). Of the 16 mice with *p53*^-/-^ genotype, 3 were wild type and 13 were heterozygous for *Diexf*^∆26^. These results clearly demonstrate that loss of p53 fails to rescue the embryonic lethality due to homozygous *Diexf* loss. To evaluate the possibility of a partial rescue (delayed lethality) upon *p53* loss in *Diexf* null embryos, we also screened embryos from *Diexf*^+/∆26^*; p53*^+/-^ double heterozygous mice crossed with *Diexf*^+/∆26^*; p53*^-/-^ animals at various time points. We failed to find any embryos with the *Diexf*^∆26/∆26^ genotype irrespective of p53 status at the time points examined, and empty decidua were observed at E7.5 and E9.5 as previously observed with the *Diexf*^+/∆26^ heterozygous crosses (Table 3). These results indicate that embryonic lethality in mice due to *Diexf* loss is not a result of inappropriate activation of the p53 pathway.

**Table 3:** Loss of p53 fails to rescue the embryonic lethality in *Diexf* null mice

D*iexf*^*+/∆26*^*; p53*^*+/−*^ *X Diexf*^*+/∆26*^*; p53*^*+/−*^ : Observed (Expected)									
Age	*Diexf*^+/+^ *p53*^+/+^	*Diexf*^*+/+*^ *p53*^+/−^	*Diexf*^+/+^ *p53*^−/−^	*Diexf*^+/−^ *p53*^+/+^	*Diexf*^+/−^*p53*^+/−^	*Diexf*^+/−^ *p53*^−/−^	*Diexf*^−/−^ *p53*^+/+^	*Diexf*^−/−^ *p53*^+/−^	*Diexf*^−/−^ *p53*^−/−^
P21	7 (7)	26 (14)	3 (7)	20 (14)	38 (27)	14 (14)	0 (7)	0 (14)	0 (7)

*Diexf*^+/∆26^*; p53*^−/−^ *X Diexf*^+/∆26^*; p53*^+/−^ : Observed (Expected)							
Age	*Diexf*^*+/+*^ *p53*^*−/+*^	*Diexf*^*+/+*^ *p53*^*−/−*^	*Diexf*^*+/−*^ *p53*^*+/−*^	*Diexf*^*+/−*^ *p53*^*−/−*^	*Diexf*^*−/−*^ *p53*^*+/−*^	*Diexf*^*−/−*^ *p53*^*−/−*^	Empty Decidua
E17.5	3 (2)	1 (2)	6 (4)	4 (4)	0 (2)	0 (2)	0
E9.5	3 (3)	3 (3)	9 (6)	8 (6)	0 (3)	0 (3)	5
E7.5	3 (2)	0 (2)	6 (4)	7 (4)	0 (2)	0 (2)	4

Inter-cross between *Diexf*^*+/∆26*^; *p53*^*+/−*^ double heterozygous animals and observed vs expected numbers for all genotypes. Bottom. Observed vs expected numbers for all genotypes in embryos derived from *Diexf*^*+/∆26*^; *p53*^*+/−*^ mice crossed with *Diexf*^*+/∆26*^; *p53*^*−*/−^ mice. All expected numbers are rounded to the nearest integer.

### *Diexf* heterozygous animals are resistant to 6Gy ionizing radiation

**Figure 3:**
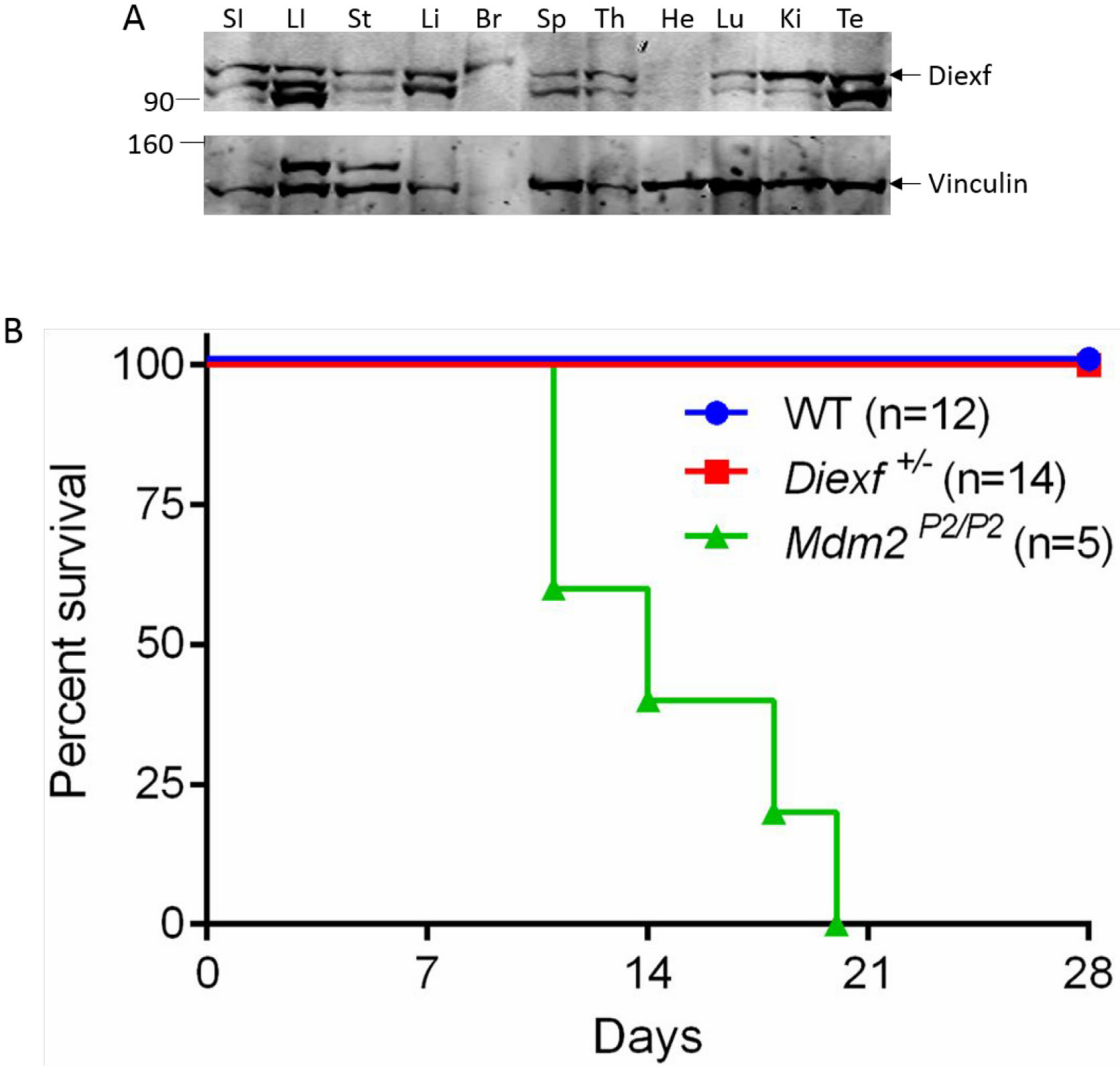
*Diexf* heterozygous animals are not sensitive to sub-lethal ionizing radiation (**A**) Western blot showing Diexf expression in different mouse tissues. Vinculin was used to evaluate protein loading. SI, Small intestine; LI, Large Intestine; St, Stomach; Li, Liver; Br, Brain; Sp, Spleen; Th, Thymus; He, Heart; Lu, Lung; Ki, Kidney; Te, Testes. (**B**) Survival curve of *Diexf*^*+/∆26*^ animals after 6 Gy radiation. Wild type and *Mdm2*^*P2/P2*^ animals were used as negative and positive controls respectively.

We next wanted to explore the possibility that *Diexf* has different functions during development and in adult animals. To test whether Diexf is expressed in adults, we first examined Diexf protein expression in various tissues of 8 week old wild type mice by immunoblotting. We observed that Diexf was expressed in all mouse tissues that we examined, with high expression in the digestive organs and low expression in the heart (Figure 3A). Next, we wanted to evaluate the role of Diexf in p53 regulation under stress condition. p53 is stabilized and activated after exposure to stress conditions such as DNA damage [2]. Previous studies in our lab have shown that heterozygosity of *Mdm2* and *Mdm4* results in sensitivity to sub-lethal ionizing radiation (IR) in a p53-dependent manner [26]. To evaluate if Diexf regulates p53 activity in the adult mouse in response to DNA damage, we irradiated 5–9 week old *Diexf*^+/∆26^ heterozygous animals (*n* = 14) along with *Mdm2*^*P2/P2*^ animals (*n* = 5) as a positive control [27], and wild type animals (*n* = 12) as a negative control. Mice were irradiated with sub-lethal IR (one dose of 6 Gy) and monitored daily for four weeks. As expected, radio-sensitive *Mdm2*^*P2/P2*^ animals died within three weeks after irradiation (Figure 3B). However, both *Diexf* heterozygous and wild-type mice survived the sub-lethal 6 Gy IR exposure and were alive by the end of 4 weeks monitoring period (Figure 3B). These results demonstrate that Diexf is not a potent negative regulator of p53 following DNA damage in adult mice.

## DISCUSSION

Understanding the mechanisms regulating the p53 pathway has important implications in cancer. MDM2 and MDM4 are critical TP53 inhibitors whose genetic loci are amplified in several tumor types that retain wild-type *TP53*. This knowledge has spurred the development of MDM2 inhibitors for cancer therapeutics. Recently, a novel gene *Diexf* was found to regulate the expression of a specific p53 isoform in zebrafish, and *p53* loss was able to partially rescue the developmental defects in the mutant zebrafish digestive system. These data suggested a potential role of Diexf in regulating the p53 pathway in mammals.

Recent studies show that Diexf homologs in *S. cerevisiae* (Utp25p) and *A. thaliana* (Nof1) are nucleolus-localized components of small subunit processome that regulate pre-rRNA processing [16, 18, 28]. In addition to this function, Def also negatively regulates p53 activity in zebrafish via proteasome-independent degradation of p53 protein in the nucleolus [15, 17, 29]. Here, we first highlighted a significant mutually exclusive relationship between *DIEXF* amplification and *TP53* mutation in human breast cancers. This relationship must, however, be considered in the context of the *DIEXF* genomic locus. *DIEXF* is in close proximity to *MDM4* gene which encodes a potent p53-inhibitor that is frequently amplified in human cancers [30, 31]. Given the data presented herein, it is likely that this mutually exclusive relationship between *DIEXF* and *TP53* is driven by amplification of *MDM4*. We cannot, however, exclude the possibility that overexpression of DIEXF contributes to p53 pathway attenuation in these cancers.

To directly evaluate the role of Diexf as a regulator of the p53 pathway in mammals, we used CRISPR/Cas9 technology to generate multiple independent *Diexf* knockout alleles in the mouse. The CRISPR/Cas9 system has revolutionized the way we generate animal models as it is efficient, fast, and less expensive than traditional techniques. One limitation of this system, however, is the difficulty in generation of knock-out alleles of genes that may be essential. If both alleles of an essential gene are disrupted, the embryos may not survive. Using only sgRNA1 and *spCas9* endonuclease, we failed to generate any mice with disruption of *Diexf* gene. We could not determine whether the sgRNA failed to target the gene or if the sgRNA was so efficient that bi-allelic disruption of the gene in all targeted mice led to lethality. When both sgRNA1 and sgRNA2 were co-injected, only sgRNA2 generated mutations at its target site indicating that sgRNA1 failed. Moreover, even though both alleles were targeted by sgRNA2 in many animals, one or both alleles were always in-frame resulting in embryo survival. If most mutations had not been in-frame, more embryos would have died. We observed the same 9 bp deletion in 10/16 mice. The observation that non-homologous end joining (NHEJ) based repair can result in frequent recurrence of the same mutation in targeted alleles also presents a problem. If the recurring mutation is frame-shift, then more embryos would die. On the other hand, if the recurring mutation is in-frame, the possibility of getting a knock-out allele is diminished. Therefore, both lethality and sgRNA failure should be considered while targeting a possible essential gene. Another major concern of the CRISPR/Cas9 system is the modifications (insertions or deletions) at off-target sites. To our knowledge, there has been no report of high frequency targeting at non-targeted sites with 2 or more mismatches in mouse models. When we screened the top four and the top six predicted off-target sites for sgRNA1 and sgRNA2 respectively, we did not find any mutations.

Using two independent null-alleles, we demonstrate that homozygous loss of *Diexf* is peri-implantation lethal, establishing *Diexf* as an essential developmental gene in the mouse. Our observation that *Diexf*-null mouse embryos are peri-implantation lethal is very different from the phenotype observed in zebrafish. In fish, the lethality occurs due to digestive organ failure, while in mice, the embryos died before any of the digestive organs develop. In addition, the abnormalities in mutant fish are p53-dependent, as the phenotype can be partially rescued by concomitant *p53* loss. However, embryonic lethality in mice due to *Diexf* loss was not rescued or even delayed by loss of *p53* demonstrating that the phenotype is not a consequence of inappropriate activation of p53. The role of p53 regulators such as Mdm4 seem to have evolved from fish to mammals as well. A recent study has shown that Mdm4 is dispensable in zebrafish, while it is essential for murine embryonic development [4, 5, 7, 25]. We could hypothesize that as the role of Mdm4 to inhibit p53 became more potent in mammals, the role of other p53 regulators such as Diexf have diminished. We cannot rule out the possibility that Diexf may cooperate with other p53 regulators to attenuate the p53 pathway, but we can conclude that it does not have an essential role in p53 regulation on its own.

Finally, we find no evidence of Diexf as a significant functional regulator of the p53 pathway in response to ionizing radiation. Heterozygous loss of well-established p53 regulators results in sensitivity to a sub-lethal dose of ionizing radiation [26]. *Diexf* heterozygous animals do not exhibit enhanced radio-sensitivity, further supporting the argument that Diexf is not a potent inhibitor of the p53 pathway in mice under these conditions. In human cancers, negative regulators of the TP53 pathway are frequently overexpressed, so it remains possible that the overexpression of DIEXF may impact the TP53 pathway and have pathological consequences. Moreover, it may inhibit TP53 function under certain conditions that we have not tested.

Combined, this work identifies *Diexf* as an essential developmental gene in the mouse, and suggests that the function(s) of Diexf are largely independent of a role as a negative regulator of p53. It has been clearly demonstrated that Def is a component of the ribosomal processome, and the Def-Capn3 pathway possibly regulates proteins other than p53 in the nucleolus. Two previous publications with *Capn3* deletion in mice show that the *Capn3*^-/-^ mice are viable and fertile, and their digestive organs are not affected [32, 33]. These results indicate that embryonic lethality in *Diexf*-null mice is independent of both p53 and Calpain3, and suggests that the role of Diexf has evolved to have very diverse functions. Moreover, as *DIEXF* amplifications occur in cancers, future studies to better understand its functions will be instructive and important.

## MATERIALS AND METHODS

### Gene targeting strategy

The entire sequence of exon 1 (starting from 20 bases upstream of the start codon) was used to score all possible target sites using the crispr.mit.edu tool. The two sites with highest scores were selected for targeting. Four and six possible off-target sites with a PAM sequence for sgRNA1 and sgRNA2 respectively were also selected for screening.

### sgRNA(s) synthesis by *in-vitro* transcription

All *in-vitro* transcription kits use T7 polymerase which requires a G at the transcription start site. For sgRNA1, N_20_ does not start with a G, and thus G (in parenthesis below) was added to the sgRNA. The T7 promoter sequence was underlined, and sgRNA target sites are in bold. Four random bases (GAAA) were added upstream of T7 promoter sequence to provide anchorage for T7 polymerase binding. The following sequence is the Forward oligo for the template. Reverse complement of this sequence will be the reverse oligo.

#### sgRNA1 forward oligo

GAAATTAATACGACTCACTATA(G)**ATGGGCAAACGCCGGAACCG**GTTTTAGAGCTAGAAATAGCAAGTTAAAATAAGGCTAGTCCGTTATCAACTTGAAAAAGTGGCACCGAGTCGGTGCTTTT

#### sgRNA2 forward oligo

GAAATTAATACGACTCACTATA**GCACCTCCGCGACTTCGGCGGTTTTAGAGCT**AGAAATAGCAAGTTAAAATAAGGCTAGTCCGTTATCAACTTGAAAAAGTGGCACCGAGTCGGTGCTTTT

Oligos were suspended in nuclease free water to a final concentration of 500 ng/µl. For each sgRNA, 5µg each of forward and reverse oligos (10 µl each) were mixed in 80µl nuclease-free water (100 µl total volume) and boiled for 5–10 minutes and then cooled at room temperature for 2 hrs to overnight. 200–400 ng of the prepared templates were used to synthesize sgRNAs using the MEGAshortscript Kit (Invitrogen AM1354) as per manufacturer’s protocol. sgRNAs were purified by acid phenol-chloroform extraction and ethanol precipitation as per manufacturer’s protocol (Invitrogen). sgRNAs were resuspended in 70 µl of RNase-free water and further purified by using Biospin P30 chromatography columns (#732–6223, Biorad) as per manufacturer’s protocol to remove any remaining free nucleotides. The concentration and quality of sgRNAs were determined by using Bio analyzer. If the concentration measured by Nanodrop was significantly higher than Bio analyzer reading, it indicated free nucleotide contamination. Column purification was repeated if necessary.

### Zygote injection and implantation

A final injection solution containing 10 ng/µl Cas9 mRNA (PrecisionX hspCas9 SmartNuclease mRNA, System Biosciences) and 7.5 ng/µl of each sgRNA was prepared in Tris-EDTA buffer (5 mM Tris, 0.1 mM EDTA). The final solution was injected into the pronucleus of 200–250 zygotes. The zygotes were then implanted into pseudo-pregnant mice (20–25 per animal). All injections and implantations were done in the MD Anderson Genetically Engineered Mouse Facility.

### Mouse breeding, maintenance, screening and genotyping

Mice were maintained in 100% C57BL/6 background. All mouse studies were conducted in compliance with an Institutional Animal Care and Use Committee protocol. Live mice were weaned at the age of three weeks, and ear biopsies were collected. The tissues were digested in Lysis buffer (1X PCR buffer, 1% Triton X, 250 µg/uL Proteinase K) at 55°C overnight, and heated at 95°C for 15 minutes to denature Proteinase K. 2 µL of the lysed tissue extract was used for PCR reaction to amplify 1 Kb region of the targeted site. The PCR product was gel purified and sanger-sequenced to identify any indels at the target site.

Screening primer Fwd: CGCATGCGTAGACACGCCTATG; Screening primer Rev: GCACAAGGGCAGAGATGATCAG; *Diexf*^∆26^ Genotyping primer Fwd: CGTTTCCGCTATGGGCAAACG; Diexf^∆26^ Genotyping primer Rev: CTCAACTCGGCCGGAACCAG.

### Selection and screening of off-target sites

A list of all possible off-target sites was obtained from crispr.mit.edu site for both sgRNAs. The following criteria were used to select the sites for screening:

1. All sites with less than 3 mismatches were selected; 2. Sites with 3 or more mismatches should be followed by PAM sequence; 3. Intra-genic sites were given preference over inter-genic sites; 4. Sites with higher score were selected first; 5. For sites with the same score, one with fewest mismatches was selected; 6. For sites with the same score and the same number of mismatches, the average of the mismatch positions were considered (one with lowest average was selected).

Once the sites were selected, the 23 base sequence for each site was run on BLAT tool at UCSC Genome Browser website, and the genomic sequence was obtained. Primers were designed about 500 bases upstream and 500 bases downstream of the selected sites, and the region was PCR amplified and sanger-sequenced.

sgRNA1 OTS1 Fwd: CCTCCACCCCGCTCTAATTTC; sgRNA1 OTS1 Rev: CTGCCCTTCTCCTCTGTGGATC; sgRNA1 OTS2 Fwd: GGGAAGGAAGCTCAGGGGTTAG; sgRNA1 OTS2 Rev: CCACCTTGGAATTCCGTTCTTTC; sgRNA1 OTS3 Fwd: GGAGGCAGTGAAAGATAAAG; sgRNA1 OTS3 Rev: CGGATCACTCAGTTTGAATC; sgRNA1 OTS4 Fwd: CGGGTTGCTTTGGAAAAAATACAC; sgRNA1 OTS4 Rev: CGGGCTGACCGTATTGAGGGAATC; sgRNA2 OTS1 Fwd: GGCTGTGGTAGGTGATTAC; sgRNA2 OTS1 Rev: GGTCACCAGCTAAGGAATG; sgRNA2 OTS2 Fwd: GACCCGGGTAAGAAGAAAAAG; sgRNA2 OTS2 Rev: CGGCGAAACAGACTGTTTC; sgRNA2 OTS3 Fwd: CACACAGGATGTCACATTCC; sgRNA2 OTS3 Rev: GCGAGCTGCCCTTTTAAG; sgRNA2 OTS4 Fwd: GCCATGCAACCTCCTAAG; sgRNA2 OTS4 Rev: CTCCAGGATCTTGCTTTTGG; sgRNA2 OTS5 Fwd: CTGGGACTAGTTTCTGGACTC; sgRNA2 OTS5 Rev: GCACACCCTGTATCTAACATTG; sgRNA2 OTS6 Fwd: GGGGCAGAGACAATCATG; sgRNA2 OTS6 Rev: GGCTGAGAATGGCTCAAG.

### E7.5 and E9.5 embryo dissection

Heterozygous animals were mated with each other, and plugs were examined every morning. Females positive for plug (E0.5) were euthanized 7 days (E7.5) or 9 days (E9.5) from that date. Uteri were dissected, and each decidua was separated and dissected as previously described [34]. Embryos from each decidua were collected and genotyped.

### Blastocyst harvest and genotyping

As described previously [34], 8–12 week old female mice were super-ovulated by intra-peritoneal injection of 5 IU of PMSG and 5 IU of HCG 48 hours later. Each female was set up in an individual mating cage with a male, and examined for plugs on the following morning. Plugged females were euthanized 3 days later (E3.5), and their uteri harvested. Buffer was injected into each uterine horn to flush out the blastocysts. Blastocysts were transferred into individual wells of a 96 well plate and were subjected to lysis and protein digestion with a lysis buffer. The entire sample was denatured, and used for PCR genotyping.

### Irradiation of mice

5–9 week old mice were exposed to one dose of 6 Gy IR in a cesium-137 irradiator and monitored daily for four weeks. Moribund animals were sacrificed.

### Western blot

Protein lysates were prepared by lysing tissues in SDS buffer. Protein estimation was carried out with BCA (Protein Assay kit, Pierce). Seventy micrograms of lysate was resolved on 8% SDS-PAGE and immunoblotted with antibodies against Diexf (1:500; A305–122A, BETHYL Labs) and Vinculin (1:1000; V9131, Sigma).

### Statistical analysis

Student’s *t*-tests and Kaplan-Meier survival analyses were performed using Prism 5 software (GraphPad Software). *P*-values < 0.05 were considered statistically significant.

## SUPPLEMENTARY MATERIALS FIGURE AND TABLE




