# Supplementary material for: Loss of digestive organ expansion factor (Diexf) reveals an essential role during murine embryonic development that is independent of p53
Source: Oncotarget. 2017 Oct 26;8(61):103996–4006. doi: 10.18632/oncotarget.22087 (PMC5732782; doi:10.18632/oncotarget.22087)
Supplement: Supplementary file 1 [file oncotarget-08-103996-s001.pdf]

# Loss of digestive organ expansion factor (*Diexf*) reveals an essential role during murine embryonic development that is independent of p53

## SUPPLEMENTARY MATERIALS

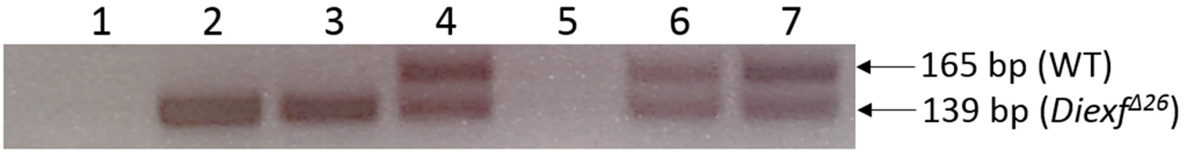


**Supplementary Figure 1: Genotyping of blastocysts.** Agarose gel electrophoresis of pcr products from blastocyst DNA showing wild type band (165 bp) and mutant band (139 bp). Lanes 2 and 3 show a single mutant band indicating *Diexf*$^{\Delta 26/\Delta 26}$ blastocysts.

**Supplementary Table 1: List of On- and off-target sites for sgRNAs.** See_Supplementary_Table 1.
